# Effect of increasing prickly pear cactus (*Nopalea cochenillifera*) levels in diets of Holstein × Zebu dairy cows on nutrients intake and digestibility, ingestive behavior, and milk fatty acids profile

**DOI:** 10.1007/s11250-025-04812-x

**Published:** 2026-03-24

**Authors:** Fernando Rossa, Adriane Pereira da Silva dos Santos, Fábio Andrade Teixeira, Claudenor de Jesus Santos, Robério Rodrigues Silva, José Esler de Freitas Júnior, Lara Maria Santos Brant, Fabiano Ferreira da Silva

**Affiliations:** 1 Department of Ruminant Production, State University of Southwestern Bahia, Itapetinga, Bahia 45.700-000 Brazil; 2 Department of Animal Science, Federal University of Bahia, Salvador, Bahia 40.170-110 Brazil

**Keywords:** Alternative forage, Cactacea, Milk production, Water use efficiency

## Abstract

This study evaluated the effects of including different levels of prickly pear cactus (*Nopalea cochenillifera*) in the diets of crossbred dairy cows (Holstein × Zebu) on nutrient intake and digestibility, nitrogen balance, microbial protein synthesis, milk yield and composition, and feeding behavior. Eight multiparous cows (15.9 ± 1.8 kg/day of milk; 100 ± 11 days in milk, mean ± SD) were assigned to a double 4 × 4 Latin square design with 21-day periods, consisting of 16 days of dietary adaptation followed by 5 days of data collection. The experimental diets were: (1) CON, without prickly pear cactus (PPC); (2) PPC16, containing 16% PPC in dry matter (DM); (3) PPC32, with 32% PPC in DM; and (4) PPC48, with 48% PPC in DM. The inclusion of PPC reduced NDF intake, both in absolute terms (*P* = 0.037) and relative to body weight (*P* = 0.021), and decreased water intake compared with the control diet (*P* < 0.001), showing a quadratic response to inclusion levels (*P* < 0.001). For digestibility, quadratic effects were observed for crude protein (*P* = 0.007) and ether extract (*P* < 0.001). Milk yield and principal components (protein, fat, and lactose) were not affected (*P* > 0.05). However, PPC reduced milk cholesterol concentration (*P* = 0.013) and altered the fatty acid profile, specifically decreasing CLA c9t11 (*P* = 0.025) and n-3 fatty acids (*P* = 0.033), while also increasing the proportion of saturated fatty acids (*P* = 0.027). It was concluded that intermediate levels of prickly pear cactus inclusion (32–35% of DM) optimized nutrient utilization while maintaining milk production, representing the most suitable supplementation strategy for crossbred dairy cows under tropical conditions.

## Introduction

The pursuit of sustainable milk production systems has intensified in recent decades, particularly in regions characterized by water scarcity and high climatic variability. In such contexts, the adoption of alternative forages capable of withstanding adverse environments while maintaining satisfactory productivity and nutritional value is essential to ensure the efficiency and viability of dairy farming (Silva et al. 2024).

Among these alternatives, the prickly pear cactus (PPC) (*Nopalea cochenillifera*) stands out due to its resilience in semiarid environments, as expressed by its remarkable drought tolerance (Ferreira et al. 2021), adaptability to low-fertility soils, high temperatures, and low rainfall (Sipango et al. 2022). Under favorable conditions, its productivity ranges from 6.3 to 17.8 tons of dry matter per hectare every two years (Costa et al. 2024), positioning it as a promising substitute for conventional forage grasses in semiarid regions.

This PPC species exhibits crassulacean acid metabolism (CAM). This physiological mechanism optimizes water use by keeping stomata closed during the day and open at night, thereby reducing transpiration (Sheoran et al. 2024). Its chemical composition further highlights its nutritional potential, with an energy value comparable to maize (Abreu Filho et al. 2022), high concentrations of non-fibrous carbohydrates (536 ± 85.1 g/kg DM), and a substantial moisture content (850–950 g/kg DM) (Siqueira et al. 2022), features that contribute to hydration and energy supply for ruminants.

Recent studies have demonstrated that the inclusion of PPC in the diets of confined dairy cows enhances feed intake due to its high palatability, water content, and wide availability in semiarid regions (Rocha Filho et al. 2021; Sobral et al. 2022). When properly balanced, it can sustain dry matter intake, nutrient digestibility, and milk production (Silva et al. 2024). Nevertheless, its low crude protein (CP) (58.60 g/kg DM) and neutral detergent fiber (NDF) concentrations (259.70 g/kg DM) (Godoi et al. 2024) limit its use as a sole feed ingredient, necessitating supplementation with fibrous and nitrogenous sources to ensure adequate ruminal fermentation (Sobral et al. 2022). Evidence further indicates that moderate inclusion levels (30–40% of dietary DM) may enhance animal performance and even improve milk fatty acid profile, reinforcing its relevance as a feed resource in semiarid dairy systems (Andrade et al. 2002; Gama et al. 2021).

Despite these advances, most research has been conducted with specialized dairy breeds (Bos taurus), and information regarding crossbred cows (Zebu × Holstein), which represent the majority of dairy herds in tropical and semiarid regions, remains scarce. These animals, better adapted to heat and forage scarcity, may benefit from the high water and energy content of forage cactus, which can help sustain dry matter intake, nutrient digestibility, and consistent milk yields under challenging environments. In hot and dry climates, the average milk production is approximately 10.38 kg/cow/day, ranging from 8.49 to 12.27 kg/cow/day (Rashid et al. 2024), and may be optimized through the strategic inclusion of forage cactus.

Therefore, the present study aimed to evaluate the effects of different levels of cactus pear (*Nopalea cochenillifera*) inclusion in the diets of crossbred dairy cows (Holstein × Zebu) on nutrient intake, digestibility, nitrogen balance, microbial protein synthesis, milk production and composition, and ingestive behavior.

## Materials and methods

### Ethical considerations and location

The study adhered to the ethical guidelines for animal use established by the Ethics Committee on Animal Use of the State University of Southwest Bahia (UESB), as approved under protocol 202/2020.

The field trial took place on a commercial farm in the municipality of Encruzilhada, Bahia, located at 15° 31′ 49″ S latitude, 40° 54′ 37″ W longitude, and an altitude of 915 m. During the experimental period, climatic conditions were characterized by temperatures ranging from 16.8 to 29 °C, with average maximum and minimum values of 27.5 °C and 17.2 °C, respectively, and an average precipitation of 95.8 mm.

### Cows, experimental design, and treatments

Eight crossbred cows (four animals with ¾ blood and four animals with ⅝ Holstein × Zebu blood), with an average milk yield of 15.9 ± 1.8 kg/day and 100 ± 11 days in milk (mean ± SD), were selected for this study. The animals were chosen from a herd with an average milk yield of 15.0 ± 2.4 kg/day, ensuring representativeness relative to the herd mean. Body condition score at calving averaged 3.0 ± 0.5 on a 1–5 scale (Edmonson et al. 1989). During the experimental period, no cows presented mastitis, and none were pregnant. All selected cows were free of complications such as dystocia, retained fetal membranes, metritis, hypocalcemia, or ketosis, ensuring uniformity and stability in the health and reproductive status of the experimental group.

The experiment followed a double 4 × 4 Latin square design, with 21-day periods consisting of 16 days for diet adaptation and management, followed by 5 days of sampling. Cows were housed in individual, covered pens measuring 16 m², each equipped with feed bunks and providing free access to feed and water. Additionally, to enhance comfort and welfare, cows had access to a covered resting corral with sand bedding one hour before the morning feeding during the experimental period.

Cows were randomly assigned to receive one of four dietary treatments: (1) CON, a diet without prickly pear cactus (PPC); (2) PPC16, inclusion of 16% PPC in the dry matter (DM); (3) PPC 32, inclusion of 32% PPC in the DM; and (4) PPC 48, inclusion of 48% PPC in the DM.

Total mixed diets were formulated according to NRC (2001) to meet the nutritional requirements of cows producing an average of 15 kg of milk/day and weighing 536 kg (Table 1). In diets containing prickly pear cactus, the inclusion of limestone and dicalcium phosphate was not necessary (Table 1), since this forage is naturally rich in calcium (Mayer and Cushman 2019), and the mineral supplement already ensured adequate Ca and P supply, preventing mineral excess and possible imbalances in the Ca: P ratio.

**Table 1:** Inclusion of ingredients and chemical composition of experimental diets

Ingredients (g/ kg of DM)	Treatments (%DM) ^a^			
	CON	PPC16	PPC 32	PPC 48
Prickly pear cactus^b^	-	159.4	317.8	479.3
Sugar cane + urea^c^	601.6	546.7	487.7	339.9
Ground corn grain	250.6	143.0	30.6	-
Soybean meal	127.9	139.4	152.4	169.3
Limestone	7.60	-	-	-
Dicalcium phosphate	0.80	-	-	-
Mineral^d^	11.5	11.5	11.5	11.5
**Chemical composition (g/kg DM)**				
Dry matter	512.5	432.3	347.9	313.9
Crude protein	160.2	163.0	165.4	167.5
Neutral detergent fiber	321.1	311.4	299.2	258.6
Ethereal extract	25.6	29.4	33.0	39.6
Ash	17.6	32.2	46.5	60.5
Non-fibrous carbohydrates^e^	455.9	452.7	444.4	462.4
iNDF^f^	156.9	165.7	173.3	160.8
Total digestible nutrients^g^	693.1	693.2	687.0	700.2
ME (Mcal/Kg DM)^h^	2.54	2.57	2.36	2.38

^a^CON = No inclusion of prickly pear cactus (PPC)in the diet, PPC16 = Inclusion of 16% prickly pear cactus in the dry matter (DM) of the diet, PPC32 = Inclusion of 32% prickly pear cactus in the DM of the diet, PPC48 = Inclusion of 48% PPC in the DM of the diet. ^b^ composition of prickly pear cactus = 131.8 g/kg of DM feed, 88.8 g/kg of DM of CP, 182.8 g/kg of DM of NDF, 59.2 g/kg of DM of EE and 561.8 g/kg of DM of NFC. ^c^ composition of sugarcane + urea = 292.2 g/kg DM, 978.9 g/kg DM of OM, 119.9 g/kg DM of CP, 456.1 g/kg DM of NDF, 14.7 g/kg DM of EE and 388.2 g/kg DM of NFC. ^d^ Composition = Calcium 200 g; Cobalt 200 mg; copper 1,650 mg; sulfur 12 g; iron 560 mg; fluorine (max) 1,000 g; phosphorus 100 g; iodine 195 mg; magnesium 15 g; manganese 1,960 mg; nickel 40 mg; selenium 32 mg; sodium 68 g and zinc 6,285 mg. ^e^ Non-fibrous carbohydrates estimated based on the Hall (2000). ^f^ Indigestible neutral detergent fiber. ^g^ Total digestible nutrients estimated by NRC (2001). ^h^ Metabolizable energy estimated by NRC (2001)

The roughage used was sugarcane (*Saccharum officinarum*), variety RB 72–454. The sugarcane was harvested three times a week, transported to a covered shed, and chopped into particles ranging from 0.5 to 1 cm in size before being offered. It was then treated with a urea and ammonium sulfate mixture in a 9:1 ratio, corresponding to 1% of the natural matter, and provided to the animals immediately after preparation. Following the recommendations of Santos et al. (2011), a 7-day adaptation period was implemented before the start of the experiment, during which the cows received the forage containing only 0.5% of the nitrogenous mixture, allowing gradual adaptation and preventing potential cases of intoxication.

The prickly pear cactus (*Nopalea cochenillifera*) was manually harvested. Only cladodes at the intermediate developmental stage were selected, while basal cladodes were preserved to maintain vegetative structure and allow new sprouting (Naorem et al. 2022). Cladodes were cut precisely at the insertion point to minimize tissue damage and reduce susceptibility to pathogen contamination. Before feeding, the material was chopped, promoting the release of mucilage, which enhanced diet homogeneity and reduced feed selectivity.

### Nutrient intake and total tract apparent digestibility

Diets were offered at 7:00 a.m. and 2:00 p.m. as a total mixed ration provided ad libitum, allowing approximately 5% orts. From days 17 to 21 of each experimental period, diet ingredients and orts samples were collected and preserved. Refusals were collected daily, and a composite sample was prepared per cow and period at the end of the collection phase. To estimate digestibility, fecal samples were collected every 9 h on days 17, 18, and 19 of each feeding period, either directly from the rectum or immediately after defecation, resulting in a total of eight samples per cow per period. Samples were immediately stored at − 20 °C, and a composite sample was later prepared for each cow at the end of each experimental phase. Fecal output was estimated using indigestible neutral detergent fiber (iNDF) as an internal marker (Detmann et al. 2012).

### Chemical analysis

Samples of diet ingredients, orts, and feces were pre-dried in a forced-air oven (55 °C for 72 h), and then they were ground using a Wiley mill with a 1 mm screen, and analyzed for dry matter (DM) content according to method 930.15; ash content, according to method 942.05; CP, following procedure 984.13; and ether extract (EE), using method 920.39, all according to the AOAC (2000) recommendations.

Samples were also analyzed for NDF (aNDFom—ash-free NDF), as described by Mertens (2002), using a fiber analyzer (Ankom Tech Corp., Fairport, NY, USA) equipped with thermostable alpha-amylase and without sodium sulfite in the detergent solution.

Non-fibrous carbohydrate (NFC) content was estimated as described by Hall (2000). The diet’s total digestible nutrients (TDN) and metabolizable energy were calculated following the NRC (2001) guidelines.

### Nitrogen balance and microbial protein synthesis

On the 17th day of each experimental period, blood samples were collected by puncturing the mammary vein and immediately centrifuged at 1,500 rpm for 15 min. The resulting plasma was stored for subsequent determination of urea concentration.

On the same day, spot urine samples were collected during spontaneous urination, four hours after feeding (11:00 a.m.). The samples were immediately filtered through gauze, and a 10 mL aliquot was diluted in 40 mL of sulfuric acid (0.036 N) to maintain the pH below 3, thus preventing microbial degradation of purine derivatives (Chen and Gomes 1992) and ammonia volatilization (Plaizier et al. 2000). The samples were then stored in plastic containers and preserved until the analysis urea, uric acid, and allantoin concentrations. A separate sample of undiluted urine (without sulfuric acid) was also preserved for total nitrogen and creatinine analyses.

Urea concentrations in lasma, urine, and deproteinized milk, as well as urinary creatinine and uric acid levels, were determined using commercial kits (Bioclin^®^, Belo Horizonte, Minas Gerais, Brazil). Allantoin concentrations were analyzed using a colorimetric method (Chen and Gomes 1992). The AOAC (2000) method 984.13 was used to determine total nitrogen in feces and urine.

Total urinary nitrogen was estimated by multiplying the nitrogen concentration (g/100 mL) in the sample by the daily urine volume. Estimation of nitrogen balance and nitrogen excretion in feces and milk followed the methodology described by Silva et al. (2021). The daily urine volume was estimated using the method described by Chizzotti et al. (2008). Nitrogen in feces, urine, and diets was also determined by the AOAC (2000) method 984.13. Urinary nitrogen excretion was calculated by multiplying urine volume (kg/day) by nitrogen concentration (g/kg).

Microbial protein synthesis was estimated based on the model proposed by Chen and Gomes (1992). The total excretion of purine derivatives (PD, mmol/day) was calculated as the sum of allantoin and uric acid present in urine and milk, according to the methodology described by Orellana-Boero et al. (2001). Microbial purine absorption (MPabs) and microbial nitrogen synthesis were calculated using the equation of Chen and Gomes (1992).

### Production, composition, and milk fatty acid profile

Cows were milked twice daily (05:00 and 17:00 h) between November and January using a mechanical milking system, with milk yield recorded throughout the experimental period by an automatic meter (MMI6^®^). Before milking, teats were disinfected with a 0.5% iodine solution (pre-dipping) and dried with disposable paper towels. Forestripping was performed to discard the first milk jets and stimulate milk letdown. After milking, the teats were immersed in a 0.5% iodine solution (post-dipping). The milking machine was inspected weekly to ensure correct vacuum pressure, pulsation rate, and hygiene procedures.

The average daily milk yield was calculated using only records obtained during the sampling phase, after the cows had adapted to the experimental diets. Milk yield was standardized to 3.5% fat-corrected milk (FCM) according to Sklan et al. (1992) and adjusted for energy-corrected milk (ECM) in accordance with NRC (2001) recommendations.

Milk samples were collected over four consecutive days (18th to 21st day of each period). Protein, fat, and lactose contents were determined using the mid-infrared method (LACTOSCAN, Entelbras^®^, São Paulo, Brazil). A second aliquot was stored at − 20 °C for cholesterol, allantoin, and urea analyses, while a third aliquot was lyophilized for subsequent determination of the milk fatty acid profile.

Allantoin and cholesterol analyses in milk were determined according to the methodologies of Chen and Gomes (1992) and Bauer et al. (2014), respectively. Urea concentrations were determined using a kit (Doles Reagentes e Equipamentos para Laboratórios Ltda., Goiânia, Brazil).

Lipids extracted from milk samples were methylated using hexane as the extraction solvent. Sodium methoxide was a catalyst, and acetyl chloride was the acidifying agent. Fatty acid methyl esters (FAMEs) were quantified using a gas chromatograph (Focus GC, Thermo Scientific, Milan, Italy) equipped with a flame ionization detector (FID) and a capillary column (SP-2560, Supelco, 100 m × 0.25 mm × 0.2 μm). The chromatographic conditions were as follows: injector and detector temperatures were set at 250 °C and 280 °C, respectively, with a split ratio of 30:1. The oven temperature was initially set at 140 °C, then increased at a rate of 1 °C/min until reaching 220 °C, where it was held for 25 min. Hydrogen was used as the carrier gas at a flow rate of 1.5 mL/min. Duplicate injections of 1 µL were performed for each extraction. The methyl esters were identified based on retention times compared to commercial fatty acid standards (GLC-674, Nu-Chek Prep, Inc.). Results were expressed as mg/100 mg of fatty acids, calculated by normalizing the peak areas.

### Eating behavior

Starting on the 20th day of each experimental phase, all cows were observed every 5 min, over a continuous period of 24 h to evaluate eating behavior (feeding, rumination, and idleness).

Observations were made on four boluses per animal using a digital stopwatch to determine the number of chews per bolus and the duration of rumination for each bolus. These measurements were taken during three distinct periods: early morning (3:00 to 5:00 a.m.), late morning to early afternoon (11:00 a.m. to 1:00 p.m.), and evening (7:00 to 9:00 p.m.). Artificial lighting was used during nighttime observations. Ingestive behavior were determined according to Bürger et al. (2000).

### Statistical analysis

The 4 × 4 double Latin square design was balanced for residual effects (Cochran and Cox 1958). The data generated were analyzed using the PROC MIXED procedure of the Statistical Analysis System software (SAS 2013) (Statistical Analysis System – SAS Institute Inc., Cary, NC, version 9.4; SAS Institute Inc., Cary, NC) using the following model:

$${Y_{ijkl}} = \mu + {\rm{ }}{S_i} + {\rm{ }}{A_i}{\left( S \right)_j} + {\rm{ }}{P_k} + {\rm{ }}{T_l} + {\rm{ }}{e_{ijkl}}$$

where Y*ijkl* is the dependent variable, µ is the overall mean, S*i* is the fixed effect of Latin square I (cow group) (i = 1–2), A*i*(S)*j* is the random effect of cow within square j (j = 1–8), P*k* is the fixed effect of experimental period k (k = 1–4), T*l* is the fixed effect of diet l (l = 1–4) and e*ijkl* is the residual error.

Orthogonal contrasts were applied to assess treatment effects, including the comparison between PPC and CON (1 1 1 − 3), as well as the linear (− 1 1 3 − 3), quadratic (− 1 − 1 1 1), and cubic (3 − 3 1 − 1) effects of PPC inclusion. Differences were considered statistically significant at *P* ≤ 0.05.

## Results

The inclusion of PPC in the diet reduced NDF intake, both in absolute values (*P* = 0.037) and relative to body weight (*P* = 0.021), compared with the CON treatment (Table 2). In contrast, EE intake was higher in the PPC treatments (*P* < 0.001), showing a quadratic effect (*P* < 0.001) with a maximum point estimated at 41.63% inclusion. TDN intake also exhibited a quadratic effect (*P* = 0.038), with a maximum point at 35.38% inclusion rate. Moreover, water intake was lower in cows fed PPC diets compared with CON (*P* < 0.001), with a quadratic response to the inclusion levels (*P* < 0.001).

**Table 2:** Intake and total-tract digestibility of dry matter and nutrients of confined lactating cows receiving inclusion levels of prickly pear cactus in the diet

Items	Treatments (%DM) ^a^				SEM^b^	*P*-value*^c^		
	CON	PPC16	PPC32	PPC48		CON x PPC	L	Q
**Intake (kg/day)**								
Dry matter	14.97	14.96	15.89	16.38	0.58	0.116	0.574	0.059
Crude protein	2.61	2.68	2.72	2.85	0.10	0.161	0.520	0.196
Neutral detergent fiber	4.94	4.94	4.96	4.51	0.17	0.037	0.093	0.230
Ether extract	0.35	0.47	0.60	0.66	0.04	< 0.001	0.740	< 0.001
Non-fibrous carbohydrates	7.14	6.69	7.11	7.45	0.26	0.145	0.237	0.190
TDN^d^	10.32	10.78	11.44	11.99	0.41	0.074	0.615	0.038
**Intake (%BW)**								
Dry matter	2.85	2.81	2.96	3.03	0.10	0.213	0.616	0.124
Neutral detergent fiber	0.94	0.93	0.93	0.84	0.03	0.021	0.091	0.130
**Intake (l/day)**								
Drinking water	47.60	36.33	18.39	8.50	3.09	< 0.001	0.095	< 0.001
**Total-tract digestibility (%)**								
Dry matter	63.4	65.4	66.6	70.2	1.69	0.177	0.541	0.212
Crude protein	65.9	74.6	76.9	78.7	1.69	0.045	0.759	0.007
Neutral detergent fiber	57.1	58.9	58.6	63.3	2.38	0.324	0.533	0.507
Ether extract	61.2	66.0	63.3	68.9	1.49	< 0.001	0.740	< 0.001
Non-fibrous carbohydrates	77.4	79.3	78.7	80.7	1.05	0.330	0.643	0.489

^*^Statistical differences were considered significant at *P* ≤ 0.05

^a^ CON = No inclusion of prickly pear cactus (PPC)in the diet, PPC16 = Inclusion of 16% prickly pear cactus in the dry matter (DM) of the diet, PPC32 = Inclusion of 32% prickly pear cactus in the DM of the diet, PPC48 = Inclusion of 48% prickly pear cactus in the DM of the diet. ^b^ Standard error of the mean. ^c^ CON vs. PPC, control vs. treatment; L, linear effect of PPC; Q, quadratic effect of PPC. ^d^ Total digestible nutrients

Regarding digestibility, higher values were observed for CP (*P* = 0.045) and EE (*P* < 0.001) in PPC diets compared with CON (Table 2). Quadratic effects were also detected for CP (*P* = 0.007) and EE digestibility (*P* < 0.001), with maximum values estimated at 34.44% and minimum values around 30.11% inclusion, respectively.

Nitrogen balance revealed greater amounts of digested nitrogen in PPC diets compared with CON (*P* = 0.013), with a quadratic effect of the inclusion levels (*P* = 0.003) (Table 3). Quadratic effects were also observed for fecal N excretion (*P* = 0.045), microbial protein synthesis (*P* = 0.015), and microbial efficiency (*P* = 0.034) (Table 3).

**Table 3:** Balance of nitrogen compounds and Urea nitrogen concentrations of confined lactating cows receiving inclusion levels of prickly pear cactus in the diet

Item	Treatments (%DM) ^a^				SEM^b^	*P*-value*^c^		
	CON	PPC16	PPC32	PPC48		CON x PPC	L	Q
Nitrogen (*N*) utilization								
Ingested nitrogen (g/day)	417.6	428.5	435.9	456.3	16.42	0.161	0.520	0.196
Fecal-N (g/day)	147.4	108.7	101.4	102.0	8.83	0.240	0.466	0.045
Milk-N (g/day)	88.2	92.1	85.9	95.7	2.57	0.074	0.090	0.835
Urinary- N (g/day)	109.9	120.6	127.0	132.4	7.75	0.439	0.932	0.333
Retained-N (g/day)	72.1	107.1	121.6	126.1	10.94	0.270	0.810	0.099
Retained-N (% ing. N)	17.0	23.9	27.7	27.1	2.22	0.399	0.667	0.123
Digested-N (g/day)	270.2	319.9	334.5	354.2	13.06	0.013	0.883	0.003
Retained-N (% dig. N)	23.0	31.9	35.6	35.0	3.04	0.496	0.696	0.208
**Urea nitrogen concentrations (mg/dL)**								
Urea nitrogen	31.9	31.4	27.2	29.3	1.23	0.699	0.442	0.097
Milk urea nitrogen	27.3	23.5	23.5	23.6	1.88	0.752	0.790	0.579
**Microbial CP synthesis (g/day)**								
Microbial crude protein	1248.2	1329.5	1570.3	1402.6	71.0	0.818	0.094	0.015
**Microbial efficiency**								
Microbial CP (g/kg of TDN)	120.9	123.3	137.3	117.0.	5.63	0.071	0.901	0.034

^*^Statistical differences were considered significant at *P* ≤ 0.05

^a^ CON = No inclusion of prickly pear cactus (PPC)in the diet, PPC16 = Inclusion of 16% prickly pear cactus in the dry matter (DM) of the diet, PPC32 = Inclusion of 32% prickly pear cactus in the DM of the diet, PPC48 = Inclusion of 48% prickly pear cactus in the DM of the diet. ^b^ Standard error of the mean. ^c^ CON vs. PPC, control vs. treatment; L, linear effect of PPC; Q, quadratic effect of PPC

No significant effects were found for milk yield or composition (*P* > 0.05), except for milk cholesterol concentration, which was higher in cows fed the CON diet (*P* = 0.013) (Table 4).

**Table 4:** Production and milk composition of confined lactating cows receiving inclusion levels of prickly pear cactus in the diet

Items	Treatments (%DM) ^a^				SEM^b^	*P*-value*^c^		
	CON	PPC16	PPC32	PPC 48		CON x PPC	L	Q
**Production (kg/day)**								
Milk yield	15.3	16.1	16.1	16.2	0.82	0.452	0.775	0.279
FCM (3.5%) ^d^	18.1	18.8	19.6	19.7	1.01	0.316	0.890	0.116
Protein	0.47	0.49	0.49	0.49	0.03	0.569	0.710	0.340
Fat	0.70	0.72	0.77	0.77	00.4	0.306	0.934	0.102
Lactose	0.70	0.74	0.75	0.74	0.02	0.613	0.617	0.330
Defatted dry extract	1.28	1.35	1.35	1.35	0.08	0.560	0.717	0.335
**Composition (g/100g)**								
Protein	3.06	3.07	3.07	3.06	0.04	0.977	0.928	0.980
Fat	4.59	4.52	4.77	4.82	0.17	0.232	0.718	0.094
Lactose	4.60	4.62	4.62	4.60	0.06	0.731	0.546	0.977
Defatted dry extract	8.37	8.37	8.37	8.37	0.13	0.984	0.985	0.961
MEC (kg/day)^g^	17.6	18.3	18.9	18.9	0.98	0.343	0.858	0.134
Cholesterol (mg/100 mL)	6.39	5.72	6.00	4.62	0.04	0.013	0.098	0.115

^*^Statistical differences were considered significant at *P* ≤ 0.05

^a^ CON = No inclusion of prickly pear cactus (PPC)in the diet, PPC16 = Inclusion of 16% prickly pear cactus in the dry matter (DM) of the diet, PPC32 = Inclusion of 32% prickly pear cactus in the DM of the diet, PPC48 = Inclusion of 48% prickly pear cactus in the DM of the diet. ^b^ Standard error of the mean. ^c^ CON vs. PPC, control vs. treatment; L, linear effect of PPC; Q, quadratic effect of PPC. ^d^ Milk yield was corrected to 3.5% fat: FCM = (0.432 + 0.165 × milk fat content) × milk yield (kg/day). ^e^ Energy-corrected milk: MEC = 0.327 × milk production (kg/day) + 12.86 × fat production (kg/day) + 7.65 × protein production (kg/day)

The milk fatty acid profile was modified by the inclusion of PPC, with increases in C18:3 n3 (*P* = 0.040) and a reduction in C18:3 n6 (*P* = 0.014). Considering the levels of PPC inclusion, quadratic effects were observed for the fatty acids C18:3 n3 (*P* = 0.002) and C18:3 n6 (*P *< 0.001). Linear effects were observed for C20:0 (*P* = 0.030) (Table 5).

**Table 5:** Fatty acid profile of milk from confined lactating cows receiving inclusion levels of prickly pear cactus in the diet

Items	Treatments (%DM)^a^				SEM^b^	*P*-value*^c^		
	CON	PPC16	PPC32	PPC48		CON x PPC	L	Q
C4:0	2.69	2.60	2.61	2.93	0.20	0.530	0.568	0.759
C6:0	1.81	1.96	1.91	1.83	0.16	0.871	0.791	0.970
C8:0	1.39	1.11	1.18	1.25	0.15	0.900	0.484	0.817
C10:0	2.31	2.57	2.72	2.76	0.21	0.633	0.935	0.466
C12:0	2.84	3.04	3.53	3.57	0.26	0.464	0.977	0.242
C14:0	10.48	10.58	11.10	11.47	0.85	0.684	0.889	0.634
C16:0	38.53	36.78	42.33	47.71	3.31	0.185	0.465	0.185
C18:0	4.73	4.45	3.63	2.87	0.41	0.118	0.555	0.085
C18:1 c9	32.48	33.87	28.08	22.87	3.37	0.288	0.582	0.273
C18:1 t10	0.70	0.70	0.79	1.06	0.29	0.335	0.532	0.452
C18:2 n6	0.03	0.04	0.04	0.02	0.00	0.191	0.215	0.555
C18:2 c9 t11	0.05	0.40	0.35	0.28	0.04	0.070	0.055	0.135
C18:2 t10 c12	0.01	0.10	0.09	0.07	0.01	0.070	0.055	0.135
C18:3 n3	0.02	0.03	0.00	0.01	0.00	0.040	0.373	0.002
C18:3 n6	0.14	0.07	0.06	0.06	0.01	0.014	0.074	<0.001
C20:0	0.01	0.06	0.05	0.03	0.01	0.648	0.030	0.410
C22:0	0.00	0.02	0.01	0.01	0.00	0.367	0.292	0.178
C22:2	0.01	0.01	0.01	0.01	0.00	0.136	0.430	0.960
Others								
>C16	38.20	39.77	33.13	27.33	3.70	0.282	0.583	0.263
< C16	22.78	22.96	24.13	25.11	1.56	0.604	0.840	0.564
C16 total	39.05	37.28	42.84	48.17	5.43	0.190	0.472	0.189
C18								
C18 Unsaturated	33.43	35.20	29.40	24.38	3.45	0.320	0.598	0.304
C18 Saturated	4.73	4.45	3.63	2.87	0.41	0.118	0.555	0.085
Total								
Saturated	64.80	63.20	69.09	74.45	4.75	0.370	0.638	0.359
Unsaturated	35.22	36.81	31.02	26.16	3.49	0.333	0.619	0.312
U/S^d^	3.29	2.14	3.02	2.84	0.34	0.966	0.803	0.695

^*^Statistical differences were considered significant at *P* ≤ 0.05

^a^ CON = No inclusion of prickly pear cactus (PPC)in the diet, PPC16 = Inclusion of 16% prickly pear cactus in the dry matter (DM) of the diet, PPC32 = Inclusion of 32% prickly pear cactus in the DM of the diet, PPC48 = Inclusion of 48% prickly pear cactus in the DM of the diet. ^b^ Standard error of the mean. ^c^ CON vs. PPC, control vs. treatment; L, linear effect of PPC; Q, quadratic effect of PPC. ^d^ Unsaturated to saturated ratio

Feeding behavior was also affected. Cows in the CON group showed longer rumination time (*P* = 0.013), greater feeding efficiency for NDF (*P* = 0.037) and rumination efficiency for NDF (*P* = 0.037), as well as higher NCB (*P* = 0.002) and TRB (*P* = 0.002) (Table 6). Linear effects were detected for rumination time (*P* = 0.037), idling time (*P* = 0.038), TCT (*P* = 0.038), and NCD (*P* = 0.046). Quadratic effects were also observed for feeding efficiency of TDN (*P* = 0.039), rumination efficiency of TDN (*P* = 0.039), NCB (*P* = 0.003), and TBR (*P* = 0.001) (Table 6).

**Table 6:** Ingestive behavior in confined lactating cows receiving inclusion levels of prickly pear cactus in the diet

Item	Treatments (%DM)^a^				SEM^b^	*P*-value^c*^		
	CON	PPC16	PPC32	PPC48		CON x PPC	L	Q
Time spent (minutes/day)								
Feeding	306.9	308.1	348.1	311.9	25.58	0.595	0.111	0.142
Rumination	536.6	534.4	541.6	475.4	26.33	0.013	0.037	0.184
Idling	596.5	597.5	550.3	652.8	45.30	0.060	0.038	0.885
**Feeding efficiency**								
DM/hour(g)	3389.7	3386.4	3596.9	3707.7	249.49	0.116	0.574	0.059
NDF/hour(g)	1119.5	1118.1	1122.1	1021.6	60.67	0.037	0.093	0.230
TDN/hour(g)	2336.0	2441.3	2589.2	2714.2	156.83	0.074	0.615	0.039
**Rumination efficiency**								
DM/hour (g)	1535.5	1534.0	1629.4	1679.6	113.02	0.116	0.574	0.059
NDF/hour (g)	507.1	506.5	508.3	462.8	17.51	0.037	0.093	0.230
TDN/hour (g)	1058.2	1105.9	1172.9	1229.5	42.19	0.074	0.615	0.039
**Chewing**								
TCT (minutes/day)^d^	843.5	842.5	889.8	787.3	23.21	0.060	0.038	0.885
NBR (number/day)^e^	443.7	530.4	529.7	564.6	20.21	0.100	0.900	0.074
NCD (number/day)^f^	30,924	31,588	32,955	28,687	986	0.069	0.046	0.761
NCB (number/bolus)^g^	74.4	62.4	61.8	52.1	2.29	0.002	0.285	0.003
TBR (second/bolus)^h^	78.3	64.2	61.3	51.9	2.71	0.002	0.441	0.001
**Feeding behavior (number of periods/day)**								
Feeding	17.4	13.8	14.9	13.9	0.64	0.113	0.856	0.134
Rumination	16.6	16.4	16.6	15.8	0.43	0.446	0.554	0.727
Idling	25.4	23.8	23.4	23.9	0.55	0.795	0.475	0.340
**Time per period of feeding behavior (hour)**								
Feeding	0.30	0.38	0.40	0.39	0.02	0.426	0.339	0.072
Rumination	0.55	0.56	0.55	0.51	0.02	0.293	0.405	0.482
Idling	0.40	0.42	0.40	0.46	0.02	0.074	0.142	0.511

^*^Statistical differences were considered significant at *P* ≤ 0.05

^a^ CON = No inclusion of prickly pear cactus (PPC)in the diet, PPC16 = Inclusion of 16% prickly pear cactus in the dry matter (DM) of the diet, PPC32 = Inclusion of 32% prickly pear cactus in the DM of the diet, PPC48 = Inclusion of 48% prickly pear cactus in the DM of the diet. ^b^ Standard error of the mean. ^c^ CON vs. PPC, control vs. treatment; L, linear effect of PPC; Q, quadratic effect of PPC. ^d^ Total chewing time. ^e^ Number of boluses ruminated per day. ^f^ Number of chews per day.^g^ Number of chews per boluse.^h^ Time spent per boluse ruminated

## Discussion

The inclusion of *Nopalea cochenillifera* in the diet of crossbred lactating cows promoted significant changes in nutrient intake and digestibility, nitrogen metabolism, milk composition, and ingestive behavior, while milk yield remained stable.

One of the most consistent effects observed was the linear reduction in NDF intake and rumination time, accompanied by a decrease in the number of chews per ruminal bolus. This response is associated with the low content of physically adequate fiber and the high concentration of non-fibrous carbohydrates present in PPC (Bezerra et al. 2023). In fact, the replacement of sugarcane with forage palms continuously reduced fiber supply, which was expected due to the lower NDF content (18.8%) and, mainly, the lower physically effective fiber content in this feed. Similar results have been reported in ruminants fed PPC, where the partial replacement of conventional forages reduced rumination without affecting dry matter intake (Araújo et al. 2023; Rodrigues et al. 2023).

In addition, a quadratic effect of PPC inclusion on EE intake and digestibility was observed. The initial increase in dietary lipid content favored the utilization of this fraction; however, at higher inclusion levels, the relative excess of lipids may have altered ruminal fermentation, reducing the activity of cellulolytic microorganisms and limiting biohydrogenation (Fiorentini et al. 2015), which resulted in lower EE digestibility. Although values remained below the critical threshold of 7% of DM established by NASEM (2021), this imbalance explains the observed quadratic response.

Regarding protein metabolism, a quadratic response was observed for CP digestibility, digested nitrogen, and microbial protein synthesis, with maximum values at intermediate inclusion levels. Microbial efficiency (g MCP/kg TDN) was also maximized at moderate PPC inclusion, indicating improved nitrogen utilization, likely due to the greater availability of fermentable carbohydrates, which favors ammonia capture and microbial protein synthesis (Lu et al. 2019). Conversely, at higher PPC levels, the reduction in rumination time and fiber effectiveness may compromise ruminal stability, resulting in excess ruminal ammonia and reduced nitrogen use efficiency (Rocha Filho et al. 2021). These findings highlight the importance of synchronizing the supply of rapidly fermentable energy and nitrogen to optimize nitrogen balance (Lu et al. 2019).

The high moisture content of cactus resulted in a reduction in drinking water intake, from 47.6 to 8.5 L/d (an 82% decrease), which represents an essential advantage for production systems in semiarid regions (Lopes et al. 2020; Cordova-Torres et al. 2022). In addition to reducing the need for supplemental water, this characteristic likely influenced ruminal dynamics, affecting lipid metabolism and the availability of fatty acids for intestinal absorption. In this context, the inclusion of PPC increased C18:3 n3 concentrations and reduced C18:3 n6 in milk, with quadratic responses according to inclusion levels, suggesting selective modulation of ruminal biohydrogenation of polyunsaturated fatty acids. This effect may be associated with the fermentative characteristics of PPC and the presence of soluble compounds, which can alter ruminal microbial activity and the hydrogenation pathways of essential fatty acids. Additionally, a linear effect was observed for C20:0, indicating subtle changes in the incorporation of long-chain fatty acids into milk, possibly related to changes in the availability or post-ruminal metabolism of these compounds.

Ingestive behavior mirrored these physicochemical characteristics: the low NDF content and low physically adequate fiber reduced rumen bolus formation, decreasing rumination time and increasing idling time. There was also a linear increase in TDN use efficiency per hour of feeding and rumination, indicating greater energy utilization per unit of time, which could favor productive performance. The high proportion of rapidly fermentable carbohydrates accelerated rumen emptying, thereby reducing the time required for physical feed processing (Knupp et al. 2019; Beltrão et al. 2021; Silva et al. 2021.

## Conclusions

The inclusion of prickly pear cactus in the diet of dairy cows improved protein and ether extract digestibility, increased nitrogen metabolism efficiency, reduced water intake, and maintained milk yield and composition. A substitution level between 32 and 35% of dietary dry matter provided the best balance between nutrient utilization, productive performance, and water use efficiency, representing the most suitable feeding strategy under tropical conditions.

## Data Availability

The datasets generated during and/or analyzed during the current study are not publicly available due to [WHY DATA ARE NOT PUBLIC] but are available from the corresponding author on reasonable request.
